# Humanizing Pedophilia as Stigma Reduction: A Large-Scale Intervention Study

**DOI:** 10.1007/s10508-021-02057-x

**Published:** 2021-10-29

**Authors:** Craig A. Harper, Rebecca Lievesley, Nicholas J. Blagden, Kerensa Hocken

**Affiliations:** 1 Department of Psychology, Nottingham Trent University, 50 Shakespeare Street, Nottingham, NG1 4FQ UK; 2 Freedom Psychology Ltd, Nottingham, UK; 3 Her Majesty’s Prison and Probation Service, Nottingham, UK

**Keywords:** Pedophilia, Social attitudes, Sexual abuse prevention, Narrative humanization, Public health

## Abstract

The stigmatization of people with pedophilic sexual interests is a topic of growing academic and professional consideration, owing to its potential role in moderating pedophiles’ emotional well-being, and motivation and engagement in child abuse prevention schemes. Thus, improving attitudes and reducing stigmatization toward this group is of paramount importance. Prior research has suggested that narrative humanization—presenting personal stories of self-identified non-offending pedophiles—could be one route to doing this. However, this work has only been conducted with students or trainee psychotherapists, meaning the public generalizability of this method is still unknown. In this study, we compared two stigma interventions to test whether narratives reduce stigma toward people with pedophilic interests more effectively than an informative alternative (scientific information about pedophilia). Using a longitudinal experimental design with a lack of non-intervention control (initial *N* = 950; final *N* = 539), we found that narratives had consistently positive effects on all measured aspects of stigmatization (dangerousness, intentionality), whereas an informative alternative had mixed results, and actually increased perceptions of pedophiles’ levels of deviance. These effects were still present four months after the initial presentation. We discuss these data in relation to ongoing debates about treating pedophilia as a public health issue requiring a broad societal approach to well-being and child abuse prevention.

## Introduction

Many researchers have begun to explore sexual interests in children via sexual abuse prevention and well-being perspectives (see Elchuk et al., 2021; Lievesley & Harper, 2021; Lievesley et al., 2020; Seto, 2018). However, there is an acknowledgement within the literature that the effective treatment of individuals with such sexual interests is contingent on the availability of suitable services, the willingness of professionals to work with this client group, and the client group feeling comfortable in seeking support that is made available (Grady et al., 2019; Jahnke, 2018a, 2018b; Levenson & Grady, 2019; Lievesley & Harper, 2021). As such, finding methods of effective stigma reduction is becoming an important topic of study in relation to this client group (Harper et al., 2018; Jahnke, 2018a, 2018b). In this paper, we ask whether previously observed effects of narrative humanization—the process by which stigma toward people with pedophilic sexual interests is reduced by presenting personal stories from the perspective of people within this community—are observable at scale within a large community sample, and whether they are persistent over time.

### Defining Pedophilia

Pedophilia is defined as a persistent and recurrent sexual interest in prepubertal children (Finkelhor, 1984; Schmidt et al., 2013; Seto, 2018). In the fifth edition of the *Diagnostic and Statistical Manual of Mental Disorders* (American Psychiatric Association, 2013) further defines pedophilic disorder as a persistent sexual interest in prepubescent children, which manifests itself in thoughts, fantasies, urges, sexual arousal, or sexual behavior, and is accompanied by either acting on or experiencing distress because of this interest. Pedophilia is not synonymous with sexual offending against children, though it is often conflated with child sexual abuse in popular and academic discourses (Feelgood & Hoyer, 2008). Seto (2018) emphasized that most men who sexually abuse children are not pedophiles, nor do all pedophiles sexually abuse children. Empirically, among child sexual abusers across multiple assessment approaches, a subgroup of between 20 and 50% can be classified as pedophilic (Schmidt et al., 2013).

The common principle across multiple definitions of pedophilia is that it constitutes a stable sexual preference, independent of arousability/intensity of sexual feeling or gender preference (Seto, 2012). Ahlers and Schaefer (2010) proposed that human sexual preferences should be described using three independent dimensions. The first dimension is sexual orientation, referring to sexual gender preference along the continuum of heterosexuality–bisexuality–homosexuality. The second dimension is sexual directedness, referring to age preference along a continuum of children–adolescents–adults. The third dimension is sexual inclination, referring to preferences for specific characteristics in others or specific sexual activities. Hence, a person with pedophilic sexual interests might be described as showing either a heterosexual, bisexual or homosexual orientation, depending on the sex of children preferred, with sexual directedness toward children, and any form of sexual inclination. In this conceptualization, sexual preference is therefore differentiated from sexual drive, which is defined as the intensity and frequency of sexual feelings and motivations (Pfaus, 1999). Defining sexual preference in this comprehensive way is important as having a sexual interest in children is an empirically supported risk factor for sexual recidivism (Mann et al., 2010). This means that when it is present, risk of offending is raised as sexual preference acts as an intrinsic motivator for sexual behavior (Seto, 2019). However, it is important to distinguish whether an individual is exclusively attracted to children or attracted to adults as well as children, as it appears that only preferential pedophilic interest is a risk factor for sexual recidivism in samples of men convicted of child sexual offenses (McPhail, 2018).

### Stigmatization of Pedophilia

A number of researchers have recently begun to study the social stigmatization of people with sexual interests in minors. For example, a series of studies have examined how stigmatization of this population is reflected in (cognitively oriented) perceptions of the controllability and willful choice over having pedophilic sexual interests (Imhoff, 2015; Imhoff & Jahnke, 2018; Jahnke, 2018a, 2018b), and attributions of psychopathic or predatory offending behavior (Jahnke et al., 2015a, 2015b, 2015c). Stigmatizing attitudes may be related to the popular conflation between pedophilia and child sexual abuse (Feelgood & Hoyer, 2008; Harrison et al., 2010). As stated by King and Roberts (2017), “when asked about “sex offenders” many are inclined to envision the media-proliferated stereotypical image of a violent, predatory male pedophile” (p. 72), and by extension, we might argue that when asked about “pedophiles,” many are inclined to envision a predatory child sex offender. This view is based in intuitive and heuristically based cognition that are bound up in availability and representativeness processes (Harper & Bartels, 2018; Harris & Socia, 2016), whereby highly accessible cases of “pedophiles” committing sexual offenses are associated with particular behavioral and affective responses due to the use of the pedophile label within the mainstream media (e.g., Harper & Hogue, 2017; Imhoff & Jahnke, 2018).

At the core of the stigmatization of people with pedophilic sexual interests may be mental processes related to dehumanization. A number of research teams have examined dehumanization within the context of media representations of sexual crime, which is often dominated by offenses committed against children and subsequently conflated with “pedophilia” (Feelgood & Hoyer, 2008). For example, Harper and Hogue (2015, 2017) reported how tabloid readers express more negative attitudes toward people convicted of sexual offenders than do broadsheet readers. However, the only differences in how these types of newspaper reported on sexual crime was in the headlines. Tabloids were more overtly hostile and dehumanizing in their descriptors of those convicted of sexual crimes (e.g., “beast,” “monster,” and “fiend”), whereas broadsheets were more descriptive, typically using the crime type or the perpetrators’ prior occupations. In addition to this work, Viki et al. (2012) reported how the dehumanization of people convicted of sexual offenses by the public is associated with lower levels of support for rehabilitation and reintegration, and higher levels of support for punitive public policy. Within the professional context, dehumanization by clinicians was associated with lower ratings of therapeutic alliance, which is in turn predictive of worse therapeutic outcomes (Beech & Hamilton-Giachritsis, 2005).

### The Importance of Addressing Stigmatization

It is not only in social attitudes, public policy, and treatment settings that the stigmatization of people with pedophilic sexual interests has negative effects. According to Jahnke et al. (2015a, 2015b, 2015c), pedophiles may self-stigmatize, with this having profound effects on well-being. For instance, greater levels of perceived stigmatization (operationalized as perceptions of negative views about pedophiles among the general population) were associated with increased levels of fear related to being “discovered” or “outed” as a pedophile. Such cognitions may subsequently lead to thought and identity suppression (Lievesley et al., 2020), with such active attempts at concealing one’s identity being associated with a host of negative downstream effects for mental health. These issues are important as they have significant implications for public health from both a mental well-being and child abuse prevention perspective (Cantor, 2014; Cantor & McPhail, 2016; Lievesley & Harper, 2019). From a well-being perspective, people with sexual interests in children have reported that professionals working with them appear to focus more on risk reduction (e.g., controlling sexual urges), despite them preferring to be supported in relation to the more general psychological well-being issues mentioned above (B^4^U-ACT, 2011; Blagden et al., 2018). This in turn leads many people with pedophilic sexual interests to be unwilling to come forward to access mental health support because of doubts over whether those professionals offering such services will act in non-judgmental ways (B^4^U-ACT, 2011; Jahnke, 2018a; Levenson & Grady, 2019). Indeed, evidence suggests that the stigma experienced by those who have a dominant sexual interest in children impairs help-seeking behaviors due to both perceived and anticipated rejection (Goode, 2010; Grady et al., 2019; Levenson & Grady, 2019).

Instead, these individuals may seek out a range of online fora (e.g., *Virtuous Pedophiles* and *B4U-ACT*) wherein they can communicate with and be supported by other non-offending people with pedophilic interests in a safe environment. The scale of the use of these fora suggests that the level of supply of services may be far lower than the demand for them (B^4^U-ACT, 2011; Cantor & McPhail, 2016). In identifying such a need to address stigma to improve both (1) general levels of well-being among MAPs and (2) the uptake of support services when they are desired, we now turn to methods of addressing and reducing stigma.

### Existing Methods of Stigma Reduction

Understanding stigma and reducing prejudice toward marginalized social groups is an important and much-studied topic in social psychology. According to a recent field-wide review by Paluck et al. (2021), prejudice and stigma reduction interventions appear to fall into three main theoretical groupings. The largest of these is rooted in the contact hypothesis (Allport, 1954; Pettigrew & Tropp, 2006), which proposes that prejudices can be reduced by exposing individuals to positive encounters to representative examples of out-groups. In contemporary research, such interventions are typically based on secondhand or imagined contact (for an overview, see Crisp & Turner, 2009). In practice, this involves the presentation of written or video vignettes that depict a member of an out-group, with these designed to mentally bring about the impression of a positive encounter. Prejudice is thus reduced in these studies through positive emotional responses to out-group members. The second cluster of stigma reduction interventions focus on cognitive and emotional understanding of out-groups. This method may be thought of as a traditional psychoeducation approach, wherein stereotypes are directly challenged by the presentation of information about an out-group, its characteristics, and its experiences (Vezzali, 2017). The final cluster of stigma reduction interventions relate to social categorization. Instead of challenging stereotypes or emotional responses to an out-group, this third type of intervention challenges the very classification of people as an “out-group” at all (Gaertner & Dovidio, 2000; Tajfel & Turner, 1979). They do this in one of two different ways. The first stresses the overlaps between a perceiver (typically a study participant) and an ostensible out-group (attempting to bring the out-group into the sphere of the in-group; e.g., Hall et al., 2009). The second approach asks study participants to consider the diversity of thought and experience within an out-group (attempting to break down the homogeneous view of a collective out-group; e.g., Brauer & Er-Rafiy, 2011).

The effectiveness of each type of prejudice and stigma reduction intervention appears to be relatively similar, with changes in stigma that typically correspond to a Cohen’s *d* effect size of between 0.35 and 0.40 (Griffiths et al., 2014; Paluck et al., 2021). These are not small effects, but their relatively modest size may offer an explanation as to why any observed changes in stigma tend to be limited to the study population, to the laboratory setting, or to the immediate time frame of the intervention study (Paluck et al., 2021; Thornicroft et al., 2016). That is, the standard effect size reported in much of the meta-analytic existing work on stigma and prejudice reduction reflect “light touch interventions, the long-term impact of which remains unclear” (Paluck et al., 2021, p. 533).

Nonetheless, the evidence appears to suggest that the largest changes in stigma and prejudicial attitudes stem from interventions that involve personal contact with members of out-groups (Griffiths et al., 2014). This has implications for the current study, which adopts the narrative humanization design described in Harper et al. (2018). Narrative humanization can be defined as the process by which (typically, media-driven) stereotypes about a particular social group can be broken down and replaced with more accurate messages by presenting personal stories from people who form a part of the social group under consideration. Thinking about this approach in light of Paluck et al.’s (2021) typology of stigma reduction interventions, narrative humanization in this context combines indirect contact (via the presentation of first-person stories) with an individual with pedophilic sexual interests with an informative psychoeducation angle through a discussion of both the unchosen nature of pedophilic sexual interests and the barriers to support services. As such, we believe that this approach has the potential to offer both immediate and longer-lasting attitude change when considering public views about people with pedophilic sexual interests.

### The Present Study

Owing to the widespread social condemnation and hostility directed toward people with pedophilic sexual interests, but the potential of mental health and abuse prevention schemes for reducing risk factors associated with sexual offending, it is important to establish methods to bring about changes in the responses of the general public toward this group in order to encourage people with such interests to seek help before committing a sexual offense. Previous studies have demonstrated how narrative humanization can improve the views of students (Harper et al., 2018) and clinical professionals (Jahnke et al., 2015a, 2015b, 2015c) by reversing the processes of dehumanization described previously (Harper & Hogue, 2015, 2017; Viki et al., 2012). However, these participant groups may be naturally more receptive to progressive information about people with pedophilic sexual interests and the improvement of their treatment within society (possibly due to higher levels of openness, liberalism, professional experience, or general education; Harper et al., 2017). As such, this prior work may not reflect how this type of information presentation would be received in the general lay population. Further, no studies have examined how we might improve public attitudes toward people with pedophilic sexual interests using large samples or longitudinal designs. This is the gap in the literature that we fill with the present study.

We replicated the procedure used by Harper et al. (2018) with two key alterations. In the first deviation from this original work, we used a large public sample with an equal sex split (as compared to student participants with a heavy female skew). Second, we deviated from the single testing procedure to incorporate a follow-up survey after a period of four months to establish whether any effects held beyond the initial testing time point. These adaptations allowed us to overcome the limitations of existing stigma reduction research in relation to pedophilia, as well as adhering to good practice within the broader field of prejudice reduction (see Paluck et al., 2021).

We hypothesized that both types of presentation (humanization and scientific information) would lead to reductions in negative evaluations (related to dangerousness, the perception that pedophilia is a choice, and ascriptions of deviance) and punitiveness toward pedophiles at the policy level (Hypothesis 1a). However, we predicted that these reductions would be greater in the narrative humanization condition (Hypothesis 1b). If these interventions do work in the ways previously reported, we might also expect that participants assigned to the narrative humanization video would demonstrate less negative implicit associations about pedophilia than those assigned to watch the expert-delivered scientific information (Hypothesis 2). Due to the exploratory nature of the follow-up survey, we did not make any specific predictions about whether the initial effects would still be present after four months.

## Method

### Participants

We set out to recruit a large sample of British citizens in this study. An a priori power analysis (conducted using G*Power v3.1.9.2; Faul et al. 2007) suggested that a minimum of 328 participants were required to have 95% power to detect small effects (*f* = 0.10). In order to maximize statistical power and enhance generalizability, we set a target of 1000 participants with an approximate 50:50 split between males and females. Participants were recruited using the crowdsourcing platform *Prolific*, which acts as a database of survey participants who receive small payments in return for study participation. For this project, we set up two “tasks” on the site, each of which allowed 500 participants to take part in the study. These tasks were labeled as studies seeking to investigate “views about pedophilia.” The first task was only advertised to males, while the second was only advertised to females. This enabled us to control the approximate sex split in our sample. For both tasks, inclusion criteria (in addition to sex) were a minimum age of 18 years, and British citizenship.

A total of 1221 people expressed an interest in the study by clicking on the survey link. Of these, there were no data recorded for 206 (indicative of participants trying to complete the study using an ineligible device, such as a tablet computer or smartphone). After cleaning the remaining data (i.e., removing those who failed the attention check, who did not provide a valid *Prolific* identifier code to allow for follow-up contact, or who suggested that they had seen the experimental stimuli before), our sample was comprised of 950 participants (50% female; *M*_age_ = 36.78 years, SD = 13.75).

Data collection for the follow-up phase of the project took the same form as above, but only those participants who were included in the first phase were eligible to take part. This was done by creating a “whitelist” on the *Prolific* platform by specifying the identifier codes of eligible participants. Only the 950 participants with eligible data from phase one saw the advertisement for the second phase of the study, which took the form of a single task (separate tasks for each sex were not required for this phase as the predetermined potential sample was already balanced in relation to its sex split). Of these, 798 were still active on *Prolific* at the time of data collection. We left this advertisement open for seven days. In this period, 608 participants clicked on the survey link, representing a 76% response rate. Of these, 21 entries contained no data (indicating an incompatible device). Ten further entries were blank following the presentation of the consent screen (indicative of these participants not providing their consent to take part in the follow-up survey), and 38 participants provided an identifier code that did not match any identifiers that were collected in the initial survey. This left 539 participants (51% female; *M*_age_ = 39.83 years, SD = 13.05) for analysis at phase two. This figure represents a 43% attrition rate from the initial testing phase. These final respondents were evenly divided between our two experimental conditions, and attrition rates were similar in both experimental conditions. Attrition did not significantly change the relative proportions of men and women in the final sample. There were no significant differences in average attitudinal scores (as measured at baseline) between the included participants and those who did not complete the follow-up questionnaire (all *p*s ≥ 0.177, *d*s < 0.10). However, those who did not take part in the follow-up were significantly younger (*M* = 32.77 years; SD = 13.62) than those who completed all study phases (*M* = 39.83 years, SD = 13.05), *t*(945) = 8.09, *p* < 0.001, *d* = 0.53.

All participants were informed of the content of the study for ethical reasons but were naïve to the specific aims, experimental manipulations, and hypotheses at the point of data collection. Participants were paid £1.25 for each phase of the study that they took part in.

### Measures

#### Demographics

We asked participants to provide some basic demographic information to allow us to describe our sample. We requested information about participants’ sex and age.

#### Attitudes to Sex Offenders Scale (ATS-21)

Participants completed the ATS-21 measure (Hogue & Harper, 2019) to establish their baseline levels of attitudes toward people convicted of sexual offenses. While this may not appear to be directly relevant to the current investigation, it has been found that people typically report completing the ATS-21 with “pedophiles” and/or “rapists” in mind (Harper et al., 2017). As such, we included this measure in order to use ATS-21 scores as covariates in our analyses, so as to reduce the level of error (or “noise”) in our statistical analysis and to increase power to detect an effect of our experimental manipulation. The scale contains 21 statements (e.g., “I think I would like a lot of sex offenders”), each of which are rated using a 5-point scale ranging from 0 (strongly disagree) to 4 (strongly agree). A composite score with a range of 0–84 is calculated by adding each item response together. High scores indicate more positive attitudes. The ATS-21 demonstrated excellent internal consistency in the current study (*α* = 0.93).

#### Stigma and Punitive Attitudes Scale (SPS)

The 30-item SPS (Imhoff, 2015) was used to examine participants’ perceptions and responses to people with pedophilic sexual interests.^1^ This measure was developed specifically to examine facets of stigmatization toward pedophiles. As such, the SPS contains subscales measuring views about pedophiles’ dangerousness (5 items; e.g., “Pedophiles are dangerous for children”; *M*_*α*_ = 0.73), the intentionality of pedophilic sexual interests (6 items; e.g., “Pedophilia is something that you choose for yourself”; *M*_*α*_ = 0.87), whether pedophiles are have high levels of mental or sexual deviance (six items; e.g., “Pedophiles are sick”; *M*_*α*_ = 0.55), and the extent to which respondents demonstrate punitive attitudes toward pedophiles (13 items; e.g., “Pedophiles should be preemptively taken into custody”; *M*_*α*_ = 0.91). Each item was answered using a 7-point scale, anchored from 1 (strongly disagree) to 7 (strongly agree). A composite score for each subscale is calculated by averaging item scores. High scores indicate negative views in relation to each stigma domain.

#### Video Manipulation

Two videos were sourced for use in this study. The first (the narrative video) contained a clip from the UK television documentary *The Paedophile Next Door*, which aired in 2015. In this clip, a person self-identifying as having non-exclusive pedophilic interests (“Eddie”) faces the camera to provide information about his “coming out” as pedophilic, the discovery of his own sexual orientation, and the lack of services available for people like him who would like further support to remain offense-free. The second (the informative video) showed Dr. James Cantor speaking about his research into the neurobiological basis of pedophilia as a sexual orientation (though for a critique of this approach to understanding pedophilia, see Joyal et al., 2019). In both videos, the essence of the message was the same: that pedophilia is an innate form of sexual interest or orientation, and that more services are required to help those who do not want to sexually abuse children. Both clips were approximately five minutes long and were embedded into the online survey using code gathered from *YouTube,* where the videos were hosted.

#### Unreported Questions

As part of a broader project examining social attitudes toward people with pedophilic sexual interests, we also asked a number of open-ended questions related to participants’ feelings about the definitions and causes of pedophilia, and the availability of professional support services for people with these sexual interests. We do not report on these qualitative data in this paper, as the responses collected were not of sufficient depth or richness to allow for any meaningful analysis to be conducted.

Our survey also included an exploratory single-target implicit association test (ST-IAT), which was administered using the *iatgen* applet incorporated into Qualtrics (Carpenter et al., 2019). This was designed to give us an indirect assessment of attitudes toward people with pedophilic sexual interests. However, following feedback from peer reviewers, we do not report these data here. This is for two key reasons. First, we omitted to collect baseline ST-IAT data prior to our experimental manipulation. Second, the data from the ST-IAT demonstrated poor construct validity (correlations of *r* < 0.20 with explicit measures of stigma) and temporal validity (test–retest correlations of *r* < 0.30). As such, we had a lack of confidence about the validity of any inferences that we could draw from the ST-IAT data. We report its existence here for transparency.

### Procedure: Phase 1

We placed advertisements for the study on *Prolific* in the manner described above. Potentially interested participants were able to click on the survey link to access more information before taking part. Those using a mobile device (which were not compatible with the ST-IAT that was embedded within the survey) were automatically directed to the end of the survey, which made them ineligible to participate through the *Prolific* system. Those who still wished to participate after reading the information screen were asked to affirm their consent using a tick box at the bottom of the screen.

Participants first entered their demographic information, such as to allow for the videos to be randomly allocated within the sex groups (male/female participants) via branches employed within the survey software. After this, they then completed the ATS-21, followed by the SPS subscales in the order listed above. Before being randomly allocated to one of the videos, participants were asked two open-ended questions related to (1) definitions of pedophilia and (2) the causes of these sexual interests. Participants then watched their allocated video, which was embedded within the survey to avoid the need to leave and return to the survey page. The button allowing participants to progress to the next page of the survey was hidden for the duration of the video to ensure attention was paid to its content. As an attention check, participants were asked to describe the video that they were allocated to on the next survey page. Next, participants completed the SPS measure once again, followed by the ST-IAT. The end of the survey was marked with four further open-ended questions asking about (1) any changes to their definitions of pedophilia, (2) how they think it would be like to live with pedophilic sexual interests, (3) what they think they would do if they started to have pedophilic sexual interests, and (4) whether they felt adequate social and professional services were available for those with pedophilic interests. Participants were then thanked for their time and reminded that they would be contacted via *Prolific* in four months to complete a follow-up study.

### Procedure: Phase 2

Upon accessing the survey link, participants were reminded of the study, and asked to re-affirm their consent to participate in the second stage. As we could link demographic data using *Prolific* IDs collected in phase one, these data were not requested again here. We also did not ask participants to complete the ATS-21 again, as scores on this measure have been reported to be stable over time and resistant to individual experimental influence (Hogue & Harper, 2019). We asked participants to complete the SPS measure, followed by the same ST-IAT as was used in phase one.

### Planned Analyses

We analyzed our SPS data using a 2 (Condition: Narrative vs. Informative; between-participants) × 3 (Time: Baseline vs. Immediate Change vs. Follow-Up; within-participants) mixed multivariate analysis of variance (MANOVA), with the four SPS factors (dangerousness; intentionality; deviance; punitive attitudes) as dependent variables. To replicate the procedure reported in Harper et al. (2018), we also ran this analysis with participants’ self-declared sex, age, and total ATS-21 scores as covariates in the model. However, the inclusion of these control did not alter any of the effects. As such, we only report the findings without covariation (though SPSS output files for all analyses can be accessed at https://osf.io/fa9kd/).

## Results

### Relationships Between Variables Across Time Points

We began by examining the relationships between all of our measured variables in both the narrative (Table 1) and informative (Table 2) video conditions. As indicated in each of these tables, all measures were associated with each other to a moderate or large degree in the expected directions (i.e., high scores on the SPS were associated with low scores on the ATS-21). The magnitudes of these correlations were consistent across both conditions and all three time points. Although these correlational analyses are unconnected to our main research questions, they are presented here for readers’ information.

**Table 1:** Zero-order correlations between study variables (narrative video condition; T1/T2 *n* = 480, T3 *n* = 271)

	1	2	3	4	5	6	7	8	9	10	11	12	13
1. ATS-21 (T1)	–												
2. SPS Dangerousness (T1)	− .55***	–											
3. SPS Intentionality (T1)	− .53***	.47***	–										
4. SPS Deviance (T1)	− .36***	.40***	.22***	–									
5. SPS Punitiveness (T1)	− .78***	.57***	.60***	.33***	–								
6. SPS Dangerousness (T2)	− .62***	.63***	.51***	.35***	.60***	–							
7. SPS Intentionality (T2)	− .50***	.41***	.81***	.18***	.57***	.56***	–						
8. SPS Deviance (T2)	− .41***	.34***	.22***	.72***	.35***	.45***	.22***	–					
9. SPS Punitiveness (T2)	− .75***	.49***	.60***	.33***	.91***	.68***	.64***	.42***	–				
10. SPS Dangerousness (T3)	− .55***	.65***	.43***	.30***	.51***	.67***	.40***	.36***	.51***	–			
11. SPS Intentionality (T3)	− .49***	.36***	.75***	.14*	.54***	.49***	.75***	.15*	.56***	.50***	–		
12. SPS Deviance (T3)	− .38***	.37***	.18**	.57***	.34***	.33***	.13*	.58***	.34***	.42***	.17***	–	
13. SPS Punitiveness (T3)	− .71***	.44***	.58***	.27***	.85***	.60***	.56***	.37***	.86***	.56***	.60***	.35***	–
*M*	33.73	5.38	4.08	5.12	4.34	4.61	3.73	4.92	3.88	5.11	3.78	5.00	4.09
SD	13.59	.89	1.32	.83	1.18	1.05	1.31	.79	1.22	.89	1.31	.76	1.16
*α*	.93	.61	.85	.56	.91	.77	.84	.54	.91	.69	.88	.56	.91

T1 = At baseline; T2 = Directly after video manipulation; T3 = At four-month follow-up

*p < .05, **p < .01, ***p < .001

**Table 2:** Zero-order correlations between study variables (informative video condition; T1/T2 *n* = 480, T3 *n* = 271)

	1	2	3	4	5	6	7	8	9	10	11	12	13
1. ATS-21 (T1)	–												
2. SPS Dangerousness (T1)	− .62***	–											
3. SPS Intentionality (T1)	− .52***	.51***	–										
4. SPS Deviance (T1)	− .39***	.44***	.19***	–									
5. SPS Punitiveness (T1)	− .76***	.63***	.55***	.44***	–								
6. SPS Dangerousness (T2)	− .64***	.73***	.56***	.40***	.65***	–							
7. SPS Intentionality (T2)	− .48***	.41***	.80***	.12*	.52***	.54***	–						
8. SPS Deviance (T2)	− .43***	.46***	.17***	.75***	.43***	.40***	.10*	–					
9. SPS Punitiveness (T2)	− .72***	.57***	.53***	.38***	.90***	.71***	.57***	.39***	–				
10. SPS Dangerousness (T3)	− .58***	.74***	.53***	.34***	.58**	.72***	.49***	.37***	.57***	–			
11. SPS Intentionality (T3)	− .54***	.47***	.77***	.23***	.59**	.55***	.78***	.22***	.60***	.58***	–		
12. SPS Deviance (T3)	− .43***	.43***	.26***	.60***	.36**	.34***	.21***	.58***	.31***	.39***	.25***	–	
13. SPS Punitiveness (T3)	− .71***	.58***	.49***	.40***	.87**	.68***	.54***	.37***	.88***	.63***	.64***	.36***	–
*M*	33.38	5.41	3.99	5.08	4.32	4.69	3.41	5.17	3.87	5.18	3.65	5.03	4.04
SD	13.59	.96	1.46	.86	1.20	1.10	1.34	.79	1.23	1.02	1.35	0.76	1.21
*α*	.93	.72	.90	.58	.91	.80	.86	.56	.92	.79	.89	.50	.92

T1 = At baseline; T2 = Directly after video manipulation; T3 = At four-month follow-up

*p < .05, **p < .01, ***p < .01

### Stigma and Punitive Attitudes Scale Findings

**Fig. 1:**
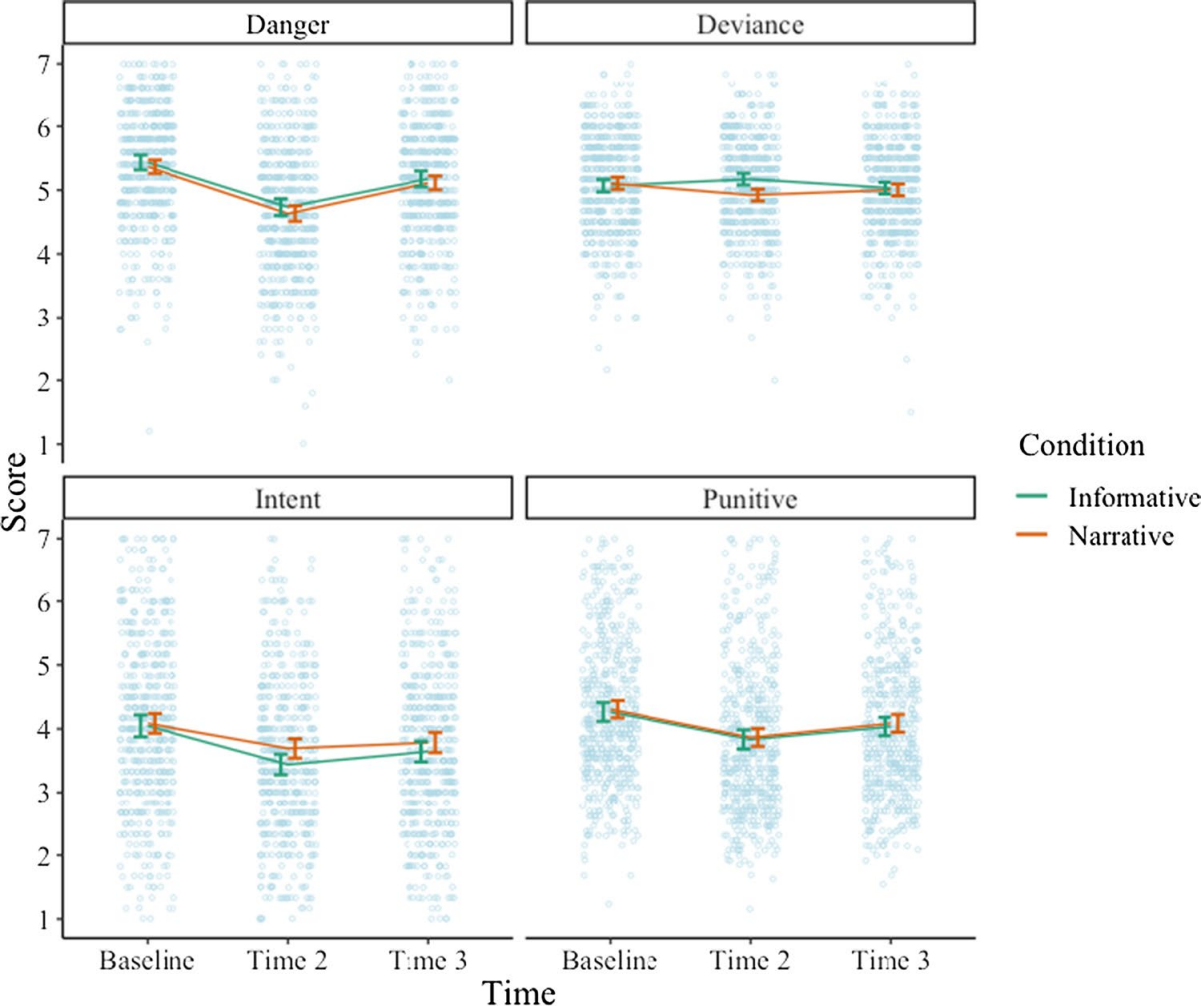
Condition × Time interactions in relation to each of the SPS factors. Error bars represent 95% CIs

Descriptive statistics for the SPS outcomes are presented in Table 3. We found a significant multivariate Condition × Time interaction (Wilk’s λ = 0.97, *F*(8, 2144) = 4.61, *p* < 0.001). Interactions for each dependent variable are depicted graphically in Fig. 1.

**Table 3:** SPS factor scores across the three time points of data collection, by condition

Condition	Dangerousness			Intentionality			Deviance			Punitiveness		
	T1	T2	T3	T1	T2	T3	T1	T2	T3	T1	T2	T3
Narrative	5.37 (.06)	4.63 (.06)	5.11 (.06)	4.09 (.08)	3.70 (.08)	3.78 (.08)	5.11 (.05)	4.92 (.05)	5.00 (.05)	4.31 (.07)	3.87 (.07)	4.09 (.07)
Informative	5.43 (.06)	4.74 (.06)	5.18 (.06)	4.05 (.08)	3.44 (.08)	3.65 (.08)	5.07 (.05)	5.17 (.05)	5.03 (.05)	4.27 (.07)	3.84 (.07)	4.04 (.07)

T1 = At baseline; T2 = Directly after video manipulation; T3 = At four-month follow-up. Data represent estimated marginal means with ± 1 *SEM* in parentheses

In relation to perceptions of pedophiles’ levels of dangerousness, there was a significant within-participants main effect of Time, *F*(2, 1074) = 248.06, *p* < 0.001, *η*^2^_*p*_ = 0.32. Examining the estimated marginal means for each time point, there was a large reduction in dangerousness perceptions immediately following the presentation of the video (*p* < 0.001, *d*_z_ = − 0.93). While these perceptions did increase again to a significant degree in the four-month follow-up (*p* < 0.001, *d*_z_ =  + 0.58), there was still a significant reduction in these beliefs at this follow-up point as compared to the initial time point (*p* < 0.001, *d*_z_ = − 0.35). The Condition × Time interaction was not significant, *F*(2, 1074) = 0.24, *p* = 0.785, *η*^2^_*p*_ < 0.001, indicating that there was no significant evidence that main effects differed across the experimental conditions. There was also no between-participants main effect of Condition, *F*(1, 537) = 1.05, *p* = 0.306, *η*^2^_*p*_ < 0.001.

Participants’ ratings of the intentionality of pedophilic sexual interests (i.e., whether these interests are innate, or chosen) changed over the three testing time points, *F*(2, 1074) = 91.09, *p* < 0.001, *η*^2^_*p*_ = 0.15. However, there was also a significant Condition × Time interaction, *F*(2, 1074) = 4.00, *p* = 0.019, *η*^2^_*p*_ = 0.01. This suggests different effects of each of the experimental videos on intentionality scores over time. In the narrative condition, there was an immediate significant reduction in intentionality perceptions after the presentation of the video (*p* < 0.001, *d*_z_ = − 0.44). There was subsequently a small and nonsignificant increase in perceptions that pedophiles choose their sexual interests in the four-month follow-up period (*p* = 0.315, *d*_z_ =  + 0.10). However, intentionality judgments at the end of the follow-up period were still significantly lower than those provided at the first point of testing (*p* < 0.001, *d*_z_ = 0.33). In the informative condition, we also observed an immediate significant reduction in intentionality perceptions (*p* < 0.001, *d*_z_ = − 0.66). However, this was followed by a significant rise in these judgments in the four-month follow-up period (*p* = 0.001, *d*_z_ =  + 0.23). Nonetheless, perceptions of the intentionality of pedophilic sexual interests were also significantly reduced between baseline and the end of the follow-up period (*p* < 0.001, *d*_z_ = − 0.42). There was no between-participants main effect of Condition, *F*(1, 537) = 1.84, *p* = 0.176, *η*^2^_*p*_ < 0.001.

There was a significant main effect of Time on perceptions of pedophiles’ levels of deviance, *F*(2, 1074) = 3.18, *p* = 0.042, *η*^2^_*p*_ = 0.01. However, there was also a significant Condition × Time interaction, *F*(2, 1074) = 14.02, *p* < 0.001, *η*^2^_*p*_ = 0.03. This again suggests that there are different trends in the effects of each of our experimental videos on this dependent variable. In the narrative condition, we witnessed a significant reduction in perceptions of pedophiles’ deviance immediately after the presentation of the video (*p* < 0.001, *d*_z_ = − 0.33). There was then a nonsignificant increase in these perceptions during the follow-up period (*p* = 0.175, *d*_z_ =  + 0.12). However, deviance judgments after the follow-up period were still marginally (though significantly) lower than those judgments that were made at the beginning of the study (*p* = 0.049, *d*_z_ = − 0.15). In contrast, there was a small immediate increase in perceptions of pedophilic deviance in the informative condition (*p* = 0.008, *d*_z_ = 0.18). This was then followed by a significant reduction during the four-month follow-up period (*p* = 0.003, *d*_z_ = 0.20), which led to deviance ratings being no different when measured at the beginning and end of the study (*p* = 1.00, *d*_z_ = 0.05). There was no between-participants main effect of Condition, *F*(1, 537) = 1.90, *p* = 0.169, *η*^2^_*p*_ < 0.001.

In relation to punitive attitudes toward pedophiles, there was a significant main effect of Time, *F*(2, 1074) = 156.86, *p* < 0.001, *η*^2^_*p*_ = 0.23. When interrogating the estimated marginal means, we observed that punitive attitudes significantly reduced immediately following the presentation of the experimental videos (*p* < 0.001, *d*_z_ = − 0.87). However, they did increase again during the follow-up period (*p* < 0.001, *d*_z_ =  + 0.35). Nonetheless, there was still a significant reduction in these attitudes between the first and third time points (*p* < 0.001, *d*_z_ = − 0.36). However, there was no significant Condition × Time interaction, *F*(2, 1074) = 0.08, *p* = 0.920, *η*^2^_*p*_ < 0.001, suggesting that the Time effects were consistent in both experimental conditions. There was also no between-participants main effect of Condition, *F*(1, 537) = 0.18, *p* = 0.676, *η*^2^_*p*_ < 0.001.

#### Overview of Stigma and Punitive Attitudes Scale Findings

For clarity, Table 4 sets out the mean differences in change scores between the conditions at each analysis point. This should be viewed as providing more inferential statistical testing of where significant differences actually occur within each respective interaction in a single tabular format.

**Table 4:** Analyses of mean differences between the experimental conditions

Outcome	Changes between T1 and T2		Changes between T2 and T3	
	*M*_difference_	Inferential test	*M*_difference_	Inferential test
Dangerousness	0.06	*t*(948) = 1.12, *p* = .262, *d* = 0.07	0.04	*t*(537) = 0.60, *p* = .551, *d* = 0.05
Intentionality	0.23	*t*(948) = 4.22, *p* < .001, *d* = 0.27	0.70	*t*(537) = 8.90, *p* < .001, *d* = 0.77
Deviance	0.29	*t*(948) = 7.45, *p* < .001, *d* = 0.48	0.22	*t*(537) = 3.69, *p* < .001, *d* = 0.32
Punitive attitudes	0.01	*t*(948) = 0.20, *p* = .840, *d* = 0.01	0.02	*t*(537) = 0.38, *p* = .701, *d* = 0.03

Mean difference scores represent differences in the amount of change between the conditions in each respective outcome between the designated time points. Please consult the descriptive data in Table 3 for directionality details. “T1” refers to baseline stigma assessments, “T2” refers to data collected immediately following the stimulus presentation, and “T3” refers to stigma scores at the four-month follow-up point

To summarize the key findings, we find similar immediate reductions in stigma, followed by gradual increases during the four-month follow-up period, in relation to perceptions of dangerousness and punitive attitudes, respectively. For intentionality (perceptions of choice over pedophilic sexual interests), although both presentations led to immediate reductions in stigma, the effect was larger for those in the informative condition. However, there was a significant difference in stigma change during the follow-up period, with a significant rebound effect being observed among those in the informative condition, but not the narrative condition. In relation to perceptions of deviance (the view that pedophilia is pathological and in need of treatment), there was a significant difference in stigma change between the two conditions, with stigma increasing in the informative condition, and reducing in the narrative condition. There was also a significant difference in rebound effects for deviance perceptions, with scores in the informative condition returning to baseline, and reductions remaining stable among those presented with the narrative stimulus.

## Discussion

In this study, we sought to replicate the findings of Harper et al. (2018) by testing the effects of first-person narrative humanization and expert-delivered informative presentations of evidence on public attitudes toward people with pedophilic sexual interests. Consistent with this earlier work, we found that dangerousness perceptions and punitive attitudes toward pedophiles significantly reduced following the presentation of a video. Extending prior work, these significant effects were still present (though to a lesser degree) after four months. The persistence of these attitudinal improvements is consistent with data reported by Jahnke et al. (2015a, 2015b, 2015c), who found that narrative-based presentations led to long-term reductions in stigmatization among German psychotherapists in training. Inconsistent with Harper et al. (2018), there was no significant difference in these effects between the two experimental conditions, suggesting that both narrative and informative presentations are equally effective in reducing perceptions of pedophiles’ dangerousness, and punitive attitudes toward them.

Where our data further deviated from Harper et al. (2018) related to intentionality and deviance judgments. In terms of intentionality (i.e., the view that pedophilic sexual interests are a choice on the part of the individual experiencing them), we found that while both conditions reduced such attitudes (both immediately and at the end of the four-month follow-up), these effects were much more volatile in the informative condition. That is, while the narrative presentation slightly decreased these views immediately (and this effect endured over the follow-up period), the informative presentation led to a larger immediate reduction in intentionality judgments, followed by a significant increase in them during the follow-up. With regard to deviance judgments, we also saw diverging trends between the conditions. While the narrative presentation reduced these views immediately (and this effect endured throughout the follow-up), the informative condition led to an immediate increase in deviance judgments, followed by a subsequent decrease to bring these attitudes back to their baseline level. These data are generally consistent (though with some minor deviations) with Hypothesis 1a, but not with Hypothesis 1b. We observed no effect of our experimental manipulation on implicit valence associations with the “pedophile” label, which was not consistent with Hypothesis 2.

### Interpretation of Findings

#### How Does Narrative Humanization Work?

As indicated in the introduction to this paper, the stigmatization of people with pedophilic sexual interests may be based in processes related to dehumanization. That is, media representations of the perpetrators of child sexual abuse (and, by extension, “pedophiles,” such is the language used by many media outlets; Feelgood & Hoyer, 2008; Harper & Hogue, 2017; Imhoff, 2015) are accompanied by descriptors that depict them as monstrous and predatory. In doing so, media outlets create a dichotomy of “us” (non-pedophiles) vs. “them” (pedophiles), wherein there are differences in the core moral characters, personality traits, and behavioral dispositions between the two groups. This leads to a sense of moral outrage related to pedophiles’ sexual interests in children and the behavioral connotations that are linked to these interests, which is characterized by feelings of fear, hatred, loathing, and disgust (Bastian et al., 2013). These feelings make it much easier for those experiencing them to sanction retributive and punitive policies, such as preventative incarceration and lay suggestions for mandated chemical castration (Jahnke et al., 2015a, 2015b, 2015c).

What these presentations do is remove the sense of humanity from people with pedophilic sexual interests and reduces them to these interests and the associated behavioral implications. A narrative presentation reverses this process by presenting these individuals as people with sexual interests in children, rather than media-constructed predators driven by them. There is a substantial body of social psychological literature suggesting that being able to take the perspective of particular [groups of] individuals can decrease levels of stigmatization toward these groups (see, e.g., Chung & Slater, 2013; Prati et al., 2015; Tompkins et al., 2015; Vescio et al., 2003). Taking the perspective of another individual is an important skill that places the perceiver “in the shoes” of those they are judging. In relation to pedophilia, there are some moves to see this form of sexual attraction as a sexual orientation (Seto, 2012). By humanizing those individuals as struggling with such preferences, perceivers may be able to identify with them on a range of indices (e.g., sexual interest choice, and control of sexual behavior) by comparing these issues with their own experience of having a non-pedophilic sexual orientation that they themselves did not choose. At its core, processes of humanization instigate this type of perspective taking and interpersonal identification.

#### Pedophiles as “Doomed to Deviance” by Informative Presentations?

Possibly the most interesting and important finding in the present dataset is that related to the different effects of narrative and informative presentations on deviance judgments made about people with pedophilic sexual interests. While the narrative presentation had the expected positive effects on such ascriptions, the informative video had an immediate negative effect (i.e., perceptions of deviance increased), before this effect subsiding through the follow-up period. One possible explanation for this is that while a narrative presentation allowed our participants to see other parts of the personality and behavior of the individual depicted in the experimental video, the informative video stressed facts about pedophilic sexual interests from a medical perspective. In doing so, the biological medicalization of these interests (e.g., the associations between pedophilia and deficiencies in cerebral white matter in areas responsible for the recognition of sexual stimuli; Cantor, 2018; Cantor et al., 2008) makes these interests appear fixed and unmalleable. This is consistent with the stigma literature in relation to mental health conditions more broadly, with the endorsement of biogenetic explanations being associated with increased desires for social distance and perceived dangerousness (Kvaale et al., 2013). It is, of course, important to constrain this argument somewhat in light of the small effect sizes that we observed, and in light of the only marginally significant *p*-value in the context of the number of comparisons conducted. However, in stigma reduction research more broadly, particularly with such an explicit intervention design, it is rare to find immediate post-intervention increases in stigmatized attitudes. As such, we tentatively suggest that informative presentations about pedophilia may have negative effects on perceptions of the levels of deviance within this population.

With a lack of options available to “treat” pedophilia with respect to changing these sexual preferences (Seto, 2012), this medicalized view has the potential to produce an attitude in lay observers that people with pedophilic sexual interests are in some way “doomed to deviance” by an unchosen and unchangeable sexual interests (Dean et al., 2009; Maruna, 2001). Promoting attributional shifts away from this fatalistic notion could bring about not only changes to social attitudes to people with pedophilic sexual interests and promote the prevention of child sexual abuse, but also could have secondary effects in terms of lowering self-stigmatization in this population (for discussions of self-stigmatization and why this is important, see Grady et al., 2019; Jahnke et al., 2015a, 2015b, 2015c; Lievesley et al., 2020). That is, this shift in attribution is not unlike the self-narrative changes in the desistance literature where people with convictions construed new selves as “good people who have done bad things” rather than “bad people who do bad things” (see Maruna, 2001).

To our knowledge, this is the first time that such an argument about perceptions of people with pedophilic sexual interests has been conceptualized in this way. This is of particular importance, because as a field there is an emerging trend of trying to communicate openly about the state of the science with regard to pedophilia in a bid to reduce stigmatization and encourage such individuals to actively seek support prior to committing sexual offenses. In light of the existing literature on the stigmatization of people with pedophilic interests (Jahnke, 2018a; Jahnke & Hoyer, 2013; Jahnke et al., 2015a, 2015b, 2015c), there is a risk that even this well-meaning scientific communication could translate into internalized stigma, with people with pedophilic sexual interests either taking on this “doomed to deviance” script, or enhancing the feeling that professionals are likely to view them through the lens of their sexual interests first, and not address broader treatment needs (B^4^U-ACT, 2011). A subsequent risk in this process is that people with pedophilic sexual interests avoid experiencing this stigma and decline to access the mental health support that they may require. As such, researchers and science communicators need to be mindful about the potential messages that can be inferred from their findings, and take steps to minimize these erroneous interpretations from being made by non-expert receivers of such findings.

#### Different Approaches, But Similar Effects?

It is important to note that although we cite different processes involved in attitudes change and stigma reduction in previous sections, the absolute effects of both of the interventions tested here are relatively similar. This is particularly the case when examining stigma scores at the four-month follow-up point. A lack of statistical interaction between Time and Condition in relation to the outcomes of “dangerousness” and “punitive attitudes” suggests that neither presentation is superior when addressing these stigma domains. For intentionality judgments (i.e., whether pedophiles choose their sexual interests) we found interesting effects. Both presentations significantly reduced stigma, though informative presentations did so to a greater degree. However, there was a significant bounce-back effect in this condition that was not observed after the narrative presentation, leading to comparative stigma changes at follow-up when compared to the initial baseline measurement. In relation to perceptions of pedophiles’ levels of deviance, the informative presentation initially increased stigma (to a small degree) before these perceptions returned to baseline after four months. However, the narrative presentation led to a significant reduction in deviance perceptions that was still present at the four-month follow-up point (albeit to a reduced degree).

What we see in these data are similar (small) effects for both types of presentation, but a more gradual return toward baseline attitudes in the narrative condition—particularly in relation to perceptions of having control or choice over sexual interests. Critical readers may cite small effect sizes as a limitation of these kinds of interventions. However, given the entrenched nature of attitudes toward individuals with pedophilic sexual interests (Jahnke, 2018a; Jahnke et al., 2015a, 2015b, 2015c) it is perhaps testament to the power of humanizing presentations that any significant effects (comparative to baseline views) were still present after four months following just a five-minute intervention. The effectiveness of such a narrative delivery may be rooted in how this method operates at a cognitive level. According to the social intuitionist approach to attitude change (Haidt, 2001), presenting facts can feel confronting to the person who is on the receiving end of an attitudinal intervention. Instead, Haidt suggests that speaking to somebody’s intuitions, and then allowing them to rationalize their own subtle attitudinal shifts, represents a more effective route to long-term attitude change. Humanizing presentations achieve this by not only presenting educational messages, but also by stressing the similarities between the participant and individuals with pedophilic sexual interests.

Consistent with established models of implicit cognition (see, e.g., Arendt & Northup, 2015), repeated exposure to messages affects implicit attitudes (analogous to intuitions), which in turn affect explicit attitudes and behavioral expressions. With the brief narrative intervention used in the present study resulting in lasting attitude change (again, albeit limited in effect), we might expect further repeated exposures to such humanizing messaging to have more profound and lasting effects in a way that informative presentations may not. More fundamentally, using narrative-based presentations is consistent with moves currently afoot within the sexological and forensic psychological research fields. That is, there is an emerging movement to adopt person-first language (see Willis, 2018) and to view individuals as whole identities, rather than being viewed purely on the basis of their sexual interests or offense histories. As such, and in spite of the comparable effect sizes in the present data, the narrative humanization approach potentially offers a more effective method to change attitudes in a profound and long-term manner, and is consistent with the philosophical direction of the field.

#### Possible Implications of Humanizing People with Pedophilic Sexual Interests

Although not directly related to the data at hand, a move toward first-person language and the humanization of people with pedophilic sexual interests is consistent with ongoing efforts to both treat and manage the emotional health of people with these sexual preferences, and also to prevent them from advancing on to acts of sexual abuse (Grady et al., 2019; Lievesley et al., 2018, 2020). That is, by seeing people with such interests as individuals at different stages of their journeys toward understanding and living with their sexual preferences, and creating services within which practitioners allow them to feel safe, it is possible to encourage active and open engagement with preventative support services (Goodier & Lievesley, 2018; Levenson & Grady, 2019). As a subsequent effect of such engagement, these services afford the opportunity to work through treatment priorities that people with pedophilic sexual interests self-report as wanting to address, such as general mental health concerns (B^4^U-ACT, 2011). Consistent with this, there have been some suggestions within the applied literature that a move toward therapies that are focused around principles of acceptance offer a promising means of engaging and helping those with pedophilic sexual interests (Hocken, 2018; Lievesley et al., 2018; Walton & Hocken, 2019). Humanization is one step toward this, and we would urge future work to look at the effects of social humanization efforts on levels of well-being among people with pedophilic sexual interests, alongside related outcomes such as comfort with seeking support (if required or desired) and levels of engagement with treatment.

#### From Attitudes to Behavior?

One conclusion that we cannot draw from these data is that using humanizing (or informative) presentations of pedophilia as a stigma reduction technique leads to reductions in actual experiences of discrimination. Our data demonstrate that participants were somewhat more likely to express lower levels of stigma after being exposed to psychoeducational about pedophilia, or after hearing a narrative from somebody with sexual interests in children. The nature of the outcomes (self-reported beliefs using the SPS; Imhoff, 2015) represent cognitive and emotional facets of stigma. However, we know that stigmatization and discrimination are also expressed at the behavioral level (Corrigan et al., 2012). Our data cannot speak to this facet of stigmatization. For example, whether reductions in attitudinal stigma leads to reduces experiences of discrimination among minor-attracted people (McPhail & Stevens, 2020), or a desire to maintain social distance from people with pedophilic sexual interests. Future studies might look to explore these outcomes more directly to establish the “real-world” effects of reductions in self-reported stigmatizing attitudes. Further, it may be fruitful to explore whether changes in self-reported stigma translate into support for community-based prevention schemes (e.g., treatment units located in local areas, or differential contributions to charities with preventative vs. punitive aims).

### Limitations and Future Directions

In this study, we observed relatively small changes in attitudes toward people with pedophilic sexual interests in response to our experimental manipulations. While this may be seen as a limitation in terms of the generalizability of the conclusions that we draw, these relatively small effects might be expected. That is, attitudes toward people with these sexual interests are deeply engrained, and not very easily changeable (Harper et al., 2018; Imhoff & Jahnke, 2018; Jahnke, 2018b). Given that we only used a brief five-minute intervention in this work, we are encouraged that even this can bring about statistically significant improvements in attitudes that persist longer than an immediate post-manipulation testing session. While we limited our method to only short videos in order to replicate past research and reduce participant attrition, future research might use longer clips (e.g., full-length documentaries) to see whether deeper or more developed arguments enhance the effects that we have observed here. Further, the use of characterization in soap operas has brought about large-scale stigma and social attitude change in relation to a range of topics, such as domestic abuse, intergroup prejudice, and mental health (Moyer-Gusé, 2008; Murrar & Brauer, 2018; Slater & Rouner, 2002). One possible action to follow from the present study could be for researchers to work with directors and television producers to embed a character with pedophilic sexual interests into such a show. Doing so, and tracking attitudes as a storyline develops, could be one way to test whether this kind of humanization could work at a mass media level over an extended period of time. The effects of such a time-extensive narrative humanization intervention could be compared to a more informative approach (e.g., a college or university course on chronophilias and human sexuality) in order to tease apart some of the issues mentioned previously.

On the topic of the videos used in the present study, we suggested above that the content of the informative video contained a discussion of the unchosen and biological nature of pedophilia. In contrast, the humanizing video presented the story of one person with pedophilic interests. Although the two videos were initially chosen in the first study to reflect the unchosen nature of pedophilia from different perspectives (Harper et al., 2018), it could be that the medicalized framing of the work of James Cantor leads to different messaging being communicated in each of the videos. Future work on the specific effects of humanization might therefore use scripted materials in both conditions (ensuring a standardized message) and manipulate only the source of the information. It may also be the case that differently scripted “humanizing” and “informative” messages could be delivered by different people (e.g., academic experts vs. individuals with lived experience of pedophilia). In doing this, future work will be able to distinguish what is actually humanizing about these stimuli—the message, or the person communicating it.

Methodologically, we used a self-selecting sample from a crowdsourcing website. This method carries inherent limitations pertaining to the representativeness of the demographic composition of the sample, and the replicability and generalizability of the results. However, we conducted an a priori power analysis to determine our minimum sample size and over-recruited on this number. When such power analyses are conducted, large samples using such crowdsourced methods do appear to replicate the effects obtained from experiments using nationally representative samples (Coppock et al., 2018). As such, we are confident in the veracity of our results. Nonetheless, preregistered replications using large demographically representative samples should be encouraged to support this claim.

Our findings in relation to the deviance subscale of Imhoff’s (2015) SPS measure are perhaps confounded to some degree by the questionable internal consistency of this subscale (that is, Cronbach’s *α* was below 0.60 at all three measurement points). This low level of consistency may be due to imprecise measurement of the construct under consideration, and the wording of the items used to measure “deviance.” For example, items such as “Pedophiles are sick” can have different connotations. On the one hand, the use of “sick” could be viewed in medical terms, and analogously to something like “illness” or “sickness.” On the other, “sick” can have moral implications, and be understood as representing associated descriptors such as “disgusting” or “immoral.” These conflations may stem from the initial translation of the SPS from German to English. Future work should look at a systematic re-validation of the structure of the SPS to both confirm its dimensionality and improve its internal consistency.

A key limitation of the present study is that we compared two stigma reduction interventions in the absence of a “no intervention” control group. As such, we cannot be certain that our observed effects are not due to a general reduction in levels of stigma about pedophilia in the population over the four months between T2 and T3. Although this is possible, this is very unlikely. Attitudes about pedophilia are deeply entrenched and based on visceral responses (Harper et al., 2018). Although no test–retest reliability data for the SPS is published in the existing literature, we know that this measure is associated with attitudes toward individuals with sexual convictions (see Harper et al., 2018) due to the popular conflation of pedophilia with child molestation (Feelgood & Hoyer, 2008; Harper & Hogue, 2017; Harrison et al., 2010; King & Roberts, 2017). The ATS-21 measure used in the present study possesses strong test–retest reliability over time (Hogue & Harper, 2019). However, our assumptions in this regard should be tested in future work through the inclusion of such a neutral control condition. In addition to having tighter experimental controls, such work would also provide evidence of the temporal stability of the SPS as a measure of stigmatized attitudes toward individuals with pedophilia.

### Conclusions

In this study, we replicated and extended previous work that found a significant positive effect of narrative humanization on attitudes and stigmatization of people with pedophilic sexual interests. We found that giving members of the lay public information of pedophilia—from both the perspective of somebody with such sexual interests and evidence delivered by an expert—had positive effects on perceptions of dangerousness, and the endorsement of punitive attitudes toward this group. However, on more nuanced indices of stigmatization—particularly perceptions of pedophiles’ deviance—only a narrative-driven presentation had consistently positive effects. We suggest that academics, activists, and policymakers might look to embed such narrative presentations about pedophilia when communicating about the important public health issue in a bid to improve the psychological well-being of people with pedophilic sexual interests and reduce the incidence of child sexual abuse.

## Data Availability

Data and statistical outputs can be accessed at https://osf.io/fa9kd/.

## Footnotes

1 As the SPS was used at three different time points in this project, we report the average α coefficient across all time points here. Specific α coefficients for each phase (and by condition) are reported in Tables 1 and 2.
